# Dissemination of Carbapenem-resistant *Klebsiella pneumoniae* clinical isolates with various combinations of Carbapenemases (KPC-2, NDM-1, NDM-4, and OXA-48) and 16S rRNA Methylases (RmtB and RmtC) in Vietnam

**DOI:** 10.1186/s12879-017-2570-y

**Published:** 2017-07-04

**Authors:** Tatsuya Tada, Mitsuhiro Tsuchiya, Kayo Shimada, Tran Thi Thanh Nga, Le Thi Anh Thu, Truong Thien Phu, Norio Ohmagari, Teruo Kirikae

**Affiliations:** 1 Department of Infectious Diseases, Research Institute, National Center for Global Health and Medicine, 1-21-1 Toyama, Shinjuku, Tokyo, 162-8655 Japan; 2 Disease Control and Prevention Center, Division of Infectious Diseases, National Center for Global Health and Medicine, Shinjuku, Tokyo, 162-8655 Japan; 3 Cho Ray Hospital, Ho Chi Minh, Vietnam; 4 Department of Microbiology, Juntendo University School of Medicine, Tokyo, 113-8421 Japan

**Keywords:** Carbapenem-resistant *Klebsiella pneumoniae*, Carbapenemase, Molecular epidemiology, MLST

## Abstract

**Methods:**

Twenty-seven clinical isolates of carbapenem-resistant *Klebsiella pneumoniae* with MICs ≥4 mg/L for imipenem or meropenem were obtained from inpatients in a hospital in Vietnam. Antimicrobial susceptibility tests and whole genome sequencing were performed. Multilocus sequence typing and the presence of drug resistant genes were determined and a maximum-likelihood phylogenetic tree was constructed by SNP alignment of whole genome sequencing data.

**Results:**

All the isolates harbored one of genes encoding carbapenemases, including KPC-2, NDM-1, NDM-4 and OXA-48. Of the isolates, 13 were resistant to arbekacin with MICs ≥256 mg/L and to amikacin with MICs ≥512 mg/L. These isolates harbored a gene encoding a 16S rRNA methylase, either RmtB or RmtC. Eighteen and 4 isolates belonged to international clones, ST15 and ST16, respectively. None of the isolates had colistin-resistant factors.

**Conclusion:**

Carbapenem-resistant *K. pneumoniae* isolates belonged to international clones spread in a medical setting in Vietnam, and that these isolates harbored genes encoding various combinations of carbapenemases and 16S rRNA methylases. This is the first report of KPC-2, NDM-4 and OXA-48 producers in a medical setting in Vietnam.

## Background

Emergence of carbapenemase-producing *Klebsiella pneumoniae* isolates has become serious problems worldwide [1]. These isolates produce several carbapenemases belonging to class A, B, and D, including KPCs, NDMs and OXA-48, respectively [2]. KPC-1 was initially found in a carbapenem-resistant strain *K. pneumoniae* 1534, which was collected in a surveillance during 1996 to 1997 in the United States hospitals [3]. NDM-1 was initially identified in *K. pneumoniae* and *Escherichia coli* in 2009 in Sweden [4]. Since then, NDM-1-producing *Enterobacteriaceae* have been reported worldwide [5]. OXA-48 was first identified in *K. pneumoniae* 11,978, which was isolated in 2001 in Turkey [6].

*K. pneumoniae* producing 16S rRNA methylase genes responsible for an extremely high level of resistance to various aminoglycosides have been increasingly reported [7]. To date, 10 types of 16S rRNA methylases, including ArmA, RmtA, RmtB, RmtC, RmtD, RmtE, RmtF, RmtG, RmtH and NpmA, have been found in clinical isolates. Of them, RmtB spread widely among various bacterial species, including *Acinetobacter baumannii*, *Enterobacteriaceae* and *Pseudomonas aeruginosa*, and RmtC spread among *Enterobacteriaceae* [7].

## Methods

### Bacterial strains and antimicrobial susceptibility

Twenty-seven *K. pneumoniae* isolates with minimum inhibitory concentrations (MICs) ≥4 mg/L for imipenem or meropenem were obtained from 27 inpatients treated at a hospital, Vietnam, from from February 2014 to April 2015. Of them, 22 isolates were from respiratory tracts, 3 from pus samples, 1 from a bile sample, and 1 from a urine sample. The isolates were phenotypically identified and species identification was confirmed by 16S rRNA sequencing. MICs were determined using the microdilution method, according to the guidelines of the Clinical Laboratory Standards Institute (M100-S25). The colistin MICs were also determined by Etest in colistin-resistant isolates evaluated by broth microdilution method.

### Detection of antibiotic-resistance genes and their genetic environments

The entire genome of each isolate was extracted by DNeasy Blood & Tissue kit (QIAGEN, Tokyo, Japan) and sequenced by MiSeq (Illumina, San Diego, CA). Sequences of drug-resistance genes, including β-lactamase encoding genes at the website (https://www.ncbi.nlm.nih.gov/pathogens/beta-lactamase-data-resources/), aminoglycoside resistance genes (aminoglycoside-acetyltransferase, −adenylyltransferase and -phosphotransferase encoding genes), colistin resistance genes (*mcr-1*, *mcr-2* and *mgrB*), registered in GenBank (http://www.ncbi.nlm.nih.gov/nuccore/) and quinolone resistance genes *gyrA* and *parC*, were determined using CLC genomics workbench version 9.0.1. Genetic environments surrounding *bla*_KPC-2_, *bla*_NDMs_ and *bla*_OXA-48_ and the genes encoding the 16S rRNA methylases were determined.

### MLST and phylogenetic analysis

Multilocus sequence types (MLSTs) were deduced as described in the protocols of the Institut Pasteur MLST (IP-MLST) (http://bigsdb.pasteur.fr/klebsiella/klebsiella.html) databases. Clonal complexes (CC) were determined by eBURST version 3 (http://eburst.mlst.net). Single nucleotide polymorphisms (SNPs) of the genome sequences of all carbapenem-resistant isolates tested were identified by comparisons with the sequence of NDM-1 producing ST15 *K. pneumoniae* PMK1, (Gen Bank accession no. CP008929), with all the reads of each isolate aligned against the PMK1 sequence using CLC Genomic Workbench version 9.0.1. SNP concatenated sequences were aligned using MAFFT (http:/mafft.cbrc.jp/alignment/server/). Phylogenetic trees were constructed from the SNP concatemers. Models and parameters used for the phylogenetic analyses were computed using j-Model Test-2.1.4. A maximum-likelihood phylogenetic tree was constructed from SNP alignment with PhyML 3.0.

### Pulsed-field gel electrophoresis and southern hybridization

The plasmids in each ST strain were extracted and pulsed-field gel electrophoresis was performed as describing previously [8]. Probes for *bla*_KPC-2_, *bla*_NDMs_ and *bla*_OXA-48_ were amplified by PCR using the primer sets as follows; KPC-F-TCGCTAAACTCGAACAGG and KPC-R-TTACTGCCCGTTGACGCCCAATCC for *bla*_KPC-2_, NDM-F- TTGGCCTTGCTGTCCTTG and NDM-R- ACACCAGTGACAATATCACCG for *bla*_NDMs_ and OXA-48-F-TGTTTTTGGTGGCATCGAT and OXA-48-R-GTAAMRATGCTTGGTTCGC for *bla*_OXA-48_, respectively. Signal detection was carried out using DIG High Prime DNA Labeling and Detection Starter Kit II (Roche Applied Science, Indianapolis, IN).

### Nucleotide sequence accession numbers

The whole genome sequences of all 27 isolates have been deposited at GenBank as accession numbers DRA005275.

## Results

### Antimicrobial susceptibility

MICs of 27 carbapenem-resistant isolates were shown in Table 1. All the isolates had MIC_50_ 8 mg/L and MIC_90_ 64 mg/L for imipenem, and MIC_50_ 8 mg/L and MIC_90_ 128 mg/L for meropenem. They were resistant to ampicillin with MICs ≥1024 mg/L, to aztreonam with MIC_50_ 512 mg/L and MIC_90_ 1024 mg/L, to ceftazidime with MIC_50_ 256 mg/L and MIC_90_ > 1024 mg/L, and to ciprofloxacin with MIC_50_ 256 mg/L and MIC_90_ 512 mg/L. Of all the isolates, 19 isolates (70%) were resistant to amikacin with MIC_50_ 1024 mg/L and MIC_90_ > 1024 mg/L. They had MIC_50_ 256 mg/L and MIC_90_ > 1024 mg/L for arbekacin, MIC_50_ 0.25 mg/L and MIC_90_ 32 mg/L to colistin, and MIC_50_ 2 mg/L and MIC_90_ 2 mg/L to tigecycline. The colistin MICs of *K. pneumoniae* isolates were significantly higher by the microdilution method than by Etest. MICs of colistin using Etest were from 0.75 to 2 mg/L (Table 1).

**Table 1:** Summary of the characteristics of the 27 *Klebsiella pneumoniae* strains, including antimicrobial resistance profiles, resistance genes and MLST

Strain	MIC(mg/L)										Carbapenemases	Size of the plasmids harboring carbapenemase encoding genes	ESBL	16S rRNA methylases	Aminoglycoside modification enzymes	Mutations in DNA gyrase		MLST
	ABK	AMK	AMP	AZT	CAZ	CIP	CST^a^	IPM	MEM	TGC						*gyrA*	*parC*	
VNC Kp05	1024	>1024	>1024	512	256	256	4 (2)	8	8	1	OXA-48	50 kbp	CTX-M-15, SHV-28, TEM-1	RmtB	AAC(6′)-Ib-cr, APH(3′)-Ia	S83Y, D87A	S80I	15
VNC Kp10	256	512	>1024	1024	512	512	32 (2)	16	4	2	OXA-48	ND	CTX-M-15, SHV-28, TEM-1	RmtB	-	S83Y, D87A	S80I	15
VNC Kp13	256	1024	>1024	512	256	256	32 (2)	8	8	2	OXA-48	ND	CTX-M-15, SHV-28, TEM-1	RmtB	AAC(6′)-Ib-cr, APH(3′)-Ia	S83Y, D87A	S80I	15
VNC Kp16	1024	>1024	>1024	512	256	512	32 (2)	8	4	2	OXA-48	50 kbp	CTX-M-15, SHV-28, TEM-1	RmtB	AAC(6′)-Ib-cr, APH(3′)-Ia	S83Y, D87A	S80I	15
VNC Kp17	512	>1024	>1024	512	128	256	4 (2)	8	8	4	OXA-48	ND	CTX-M-15, SHV-28, TEM-1	RmtB	AAC(6′)-Ib-cr, APH(3′)-Ia	S83Y, D87A	S80I	15
VNC Kp20	512	>1024	>1024	256	128	256	0.25	2	8	2	OXA-48	ND	CTX-M-15, SHV-28, TEM-1	RmtB	AAC(6′)-Ib-cr, APH(3′)-Ia	S83Y, D87A	S80I	15
VNC Kp21	256	1024	>1024	512	128	256	0.25	2	4	2	OXA-48	ND	CTX-M-15, SHV-28, TEM-1	RmtB	AAC(6′)-Ib-cr, APH(3′)-Ia	S83Y, D87A	S80I	15
VNC Kp22	1024	1024	>1024	512	256	256	16 (2)	16	16	2	OXA-48	ND	CTX-M-15, SHV-28, TEM-1	RmtB	AAC(6′)-Ib-cr, APH(3′)-Ia	S83Y, D87A	S80I	15
VNC Kp25	256	>1024	>1024	256	256	256	0.125	8	2	2	OXA-48	ND	CTX-M-15, SHV-28, TEM-1	RmtB	AAC(6′)-Ib-cr, APH(3′)-Ia	S83Y, D87A	S80I	15
VNC Kp28	512	1024	>1024	512	256	256	8 (0.75)	8	8	2	OXA-48	ND	CTX-M-15, SHV-28, TEM-1	RmtB	AAC(6′)-Ib-cr, APH(3′)-Ia	S83Y, D87A	S80I	15
VNC Kp29	1	4	>1024	512	32	32	0.125	4	4	1	KPC-2	150 kbp	CTX-M-15, TEM-1, SHV-28	-	AAC(6′)-Ib-cr	S83I	S80I	307
VNC Kp30	1	2	>1024	512	128	128	0.25	4	4	2	OXA-48	ND	CTX-M-15, SHV-28, TEM-1	-	AAC(6′)-Ib-cr, AADA16 APH(3′)-Ia	S83Y, D87A	S80I	15
VNC Kp32	512	1024	>1024	1024	256	256	8 (1)	16	8	2	OXA-48	ND	CTX-M-15, SHV-28, TEM-1	RmtB	AAC(6′)-Ib-cr, APH(3′)-Ia	S83Y, D87A	S80I	15
VNC Kp34	256	1024	>1024	512	256	256	32 (2)	8	8	1	OXA-48	ND	CTX-M-15, SHV-28, TEM-1	RmtB	AAC(6′)-Ib-cr, APH(3′)-Ia	S83Y, D87A	S80I	15
VNC Kp35	256	128	>1024	512	1024	256	16 (1)	8	8	2	OXA-48	ND	CTX-M-14, SHV-12, TEM-1	RmtB	AAC(6′)-Ib-cr, AADA1, APH(3′)-Ia	S83Y, D87A	S80I	15
VNC Kp39	>1024	>1024	>1024	32	>1024	256	0.5	8	8	1	NDM-1	100 kbp	CTX-M-27, SHV-28	RmtC	AAC(6′)-Ib-cr, AADA16	S83I	S80I	395
VNC Kp42	16	16	>1024	256	>1024	64	32 (1)	32	64	1	NDM-4	120 kbp	CTX-M-15, SHV-28, TEM-1	-	AAC(6′)-Ib-cr, AADA1, AADA16	S83F, D87A	S80I	15
VNC Kp43	1024	1024	>1024	512	256	256	0.25	4	4	2	OXA-48	ND	CTX-M-14, SHV-12, TEM-1	RmtB	AAC(6′)-Ib-cr, AADA1	S83Y, D87A	S80I	15
VNC Kp51	16	16	>1024	1024	256	64	0.25	16	4	1	OXA-48	ND	CTX-M-14, SHV-12, TEM-1	-	AAC(6′)-Ib-cr, AADA1	S83Y, D87A	S80I	15
VNC Kp54	>1024	>1024	>1024	1024	256	64	0.5	8	16	1	OXA-48	145.5 kbp	CTX-M-14, CTX-M-15, SHV-1, TEM-1	RmtB	AADA2, APH(3′)-Ia	S83F, D87N	E84K	16
VNC Kp56	>1024	>1024	>1024	512	256	64	0.25	16	16	1	OXA-48	ND	CTX-M-14, CTX-M-15, SHV-1, TEM-1	RmtB	AADA2, APH(3′)-Ia	S83F, D87N	E84K	16
VNC Kp57	>1024	>1024	>1024	64	>1024	256	0.25	64	128	0.5	NDM-4	40 kbp	CTX-M-27, SHV-11, TEM-1	RmtB	-	S83I	S80I	2353
VNC Kp68	2	4	>1024	512	256	64	0.125	4	8	2	OXA-48	50 kbp	SHV-1, TEM-1	-	AAC(6′)-Ib-cr	S83I	S80I	147
VNC Kp70	16	16	>1024	1024	>1024	256	0.125	128	256	2	NDM-4	120 kbp	CTX-M-14, CTX-M-15, SHV-1, TEM-1	-	AAC(6′)-Ib-cr, AADA1, AADA2	S83F, D87N	E84K	16
VNC Kp72	16	8	>1024	128	>1024	128	0.125	32	64	2	NDM-4	ND	CTX-M-15, SHV-28, TEM-1	-	AAC(6′)-Ib-cr, AADA1, AADA16	S83F, D87A	S80I	15
VNC Kp73	16	16	>1024	1024	>1024	512	0.125	128	256	2	NDM-4	ND	CTX-M-14, CTX-M-15, SHV-1, TEM-1	-	AAC(6′)-Ib-cr, AADA1, AADA2	S83F, D87N	E84K	16
VNC Kp77	>1024	>1024	>1024	128	>1024	256	0.25	16	64	1	NDM-1	ND	CTX-M-27, SHV-28	RmtC	AAC(6′)-Ib-cr, AADA16	S83I	S80I	395

*MIC*, minimum inhibitory concentration, *ABK* arbekacin, *AMK* amikacin, *AMP* ampicillin, *AZT* azidothymidine, *CAZ* ceftazidime, *CIP* ciprofloxacin, *CST* colistin, *IPM* imipenem, *MEM* meropenem, *TGC* tigecycline, *ESBL* extended-spectrum-lactamase, *ND* not determined

^a^MICs for colistin using Etest are given in parentheses

### Drug resistant genes

All isolates tested had a carbapenemase encoding gene, such as *bla*_KPC-2_, *bla*_NDM-1_, *bla*_NDM-4_ and *bla*_OXA-48_; and the majority had a 16S rRNA methylase encoding gene, such as *rmtB* and *rmtC* (Table 1). Of the all isolates, 19 had *bla*_OXA-48_, 5 had *bla*_NDM-4_, 2 had *bla*_NDM-1_, and 1 had *bla*_KPC-2_. Seventeen had *rmtB* and 2 had *rmtC*. These isolates also had (an) extended spectrum β-lactamase encoding gene(s), including *bla*_CTX-M-14_, *bla*_CTX-M-15_, *bla*_CTX-M-27_, *bla*_SHV-1_, *bla*_SHV-11_, *bla*_SHV-12_, *bla*_SHV-28_, *bla*_SHV-55_, and/or *bla*_TEM-1_; and aminoglycoside modification enzymes, including *aac(6′)-Ib-cr* and/or *aadA1* (Table 1). All isolates had 2 or 3 point mutations in the quinolone-resistance-determining regions of *gyrA* and *parC* (Table 1). None of the isolates harbor *mcr-1* or *mcr-2*, and our analysis did not reveal any isolates with disruption in the *mrgB* gene.

### Genetic environments surrounding genes encoding carbapenemases

**Fig. 1:**
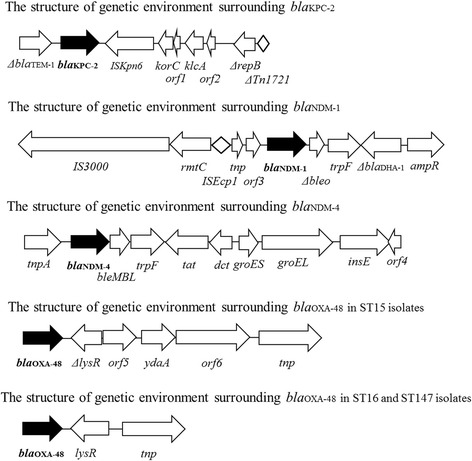
The structure of genetic environments surrounding *bla*_KPC-2_, *bla*_NDM-1_, *bla*_NDM-4_ and *bla*_OXA-48_

The genetic structure surrounding *bla*_KPC-2_, *bla*_NDM-1_, *bla*_NDM-4_ and *bla*_OXA-48_ were shown in Fig. 1. The genomic structure surrounding *bla*_KPC-2_ was identical with that of *Aeromonas hydrophila* strain WCHAH01 plasmid pKPC2 (GenBank accession no. KR014106), which was isolated in China.

All isolates harboring *bla*_NDM-1_ tested had the same genetic structure surrounding *bla*_NDM-1_ (Fig. 1), which was identical with that of plasmid pRIH26 in NDM-1 producing *K. pneumoniae* isolated from a patient in 2012 in Rhode Island, the United States [9]. This patient had returned to the United States after a hospitalization in Vietnam [9].

All isolates harboring *bla*_NDM-4_ tested had the same genetic structure surrounding *bla*_NDM-4_ (Fig. 1), which was identical to that of NDM-1 producing *K. pneumoniae* strain KP4 plasmid pKP04NDM isolated in China (GenBank accession no. KU314941).

OXA-48 producers had either one of two genetic structures surrounding *bla*_OXA-48_ (Fig. 1). Of them, one was not reported (the second structure from the bottom in Fig. 1), whereas the other was identical with plasmids in OXA-48 producing *K. pneumoniae* strains 153,877–1 in Netherlands (GenBank accession no. KP659188), KP112 in France (GenBank accession no. LN864819), Kpn-E1.Nr7 in Switzerland (GenBank accession no. KM406491), E71T in Ireland (GenBank accession no. KC335143), KP1 and KP2 in France (GenBank accession no. KC757416 and KC757417, respectively), and 23 plasmid pIncL_M_DHQP1400954 in the USA (GenBank accession no CP016927).

The *bla*_KPC-2_, *bla*_NDM-1_, *bla*_NDM-4_ and *bla*_OXA-48_ in each ST strain will be all located on plasmids and the sizes of the plasmids were shown in Table 1.

### MLST and molecular phylogenetic analysis

**Fig. 2:**
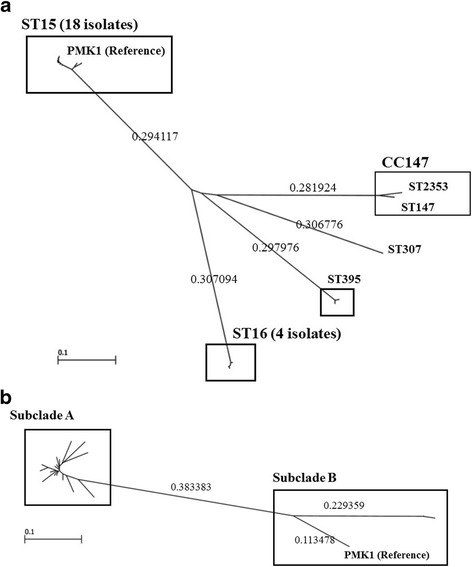
**a** Molecular phylogeny of the 27 *K. pneumoniae* strains*.* A maximum-likelihood phylogenetic tree was constructed from the 27 carbapenem-resistant isolates. The isolates belonging to ST15 formed the largest clade among the 27 isolates. **b** Molecular phylogeny of the 18 *K. pneumoniae* strains belonging to ST15*.* A maximum-likelihood phylogenetic tree was constructed from the 18 carbapenem-resistant isolates. Of them, 16 belonging to subclade A harbored *bla*_OXA-48_ and 2 belonging to subclade B harbored *bla*_NDM-4_

The clinical isolates of *K. pneumoniae* tested belonged to either one of ST15, ST16, ST147, ST307, ST395 and ST2353 (Table 1). Of these isolates, 18 belonged to ST15 and 4 belonged to ST16 (Table 1). The new sequence type, ST2353, belonged to CC147. Phylogenetic analysis revealed that the isolates belonging to ST15 formed the largest clade among the 27 isolates (Fig. 2a). The 18 isolates belonging to ST15 harbored either one of two genes encoding carbapenemases, including *bla*_OXA-48_ and *bla*_NDM-4_; and genes encoding aminoglycoside resistance factors, including *aac(6′)-Ib-cr* and *rmtB* (Table 1). The phylogenetic tree among 18 ST15 isolates showed two subclades, A and B (Fig. 1b). Of them, 16 belonging to subclade A harbored *bla*_OXA-48_ and 2 belonging to subclade B harbored *bla*_NDM-4_ (Fig. 2b).

## Discussion

To our knowledge, this is the first report of the whole genome based molecular epidemiological analysis of carbapenem-resistant *K. pneumoniae* in Vietnam. Our study suggests that carbapenem-producing ST15 *K. pneumoniae* have been spreading in medical settings in Vietnam. A NDM-1 producing *K. pneumoniae* clinical isolate in Vietnam was firstly obtained from a urinary tract of a 62-year-old man in 2010 [10].

This is the first report of NDM-4 or OXA-48 producing *K. pneumoniae* in Vietnam. NDM-4 was firstly detected in *E. coli* I5, which was recovered from a urine sample of a patient hospitalized in 2010 in India [11]. Since then, NDM-4 producers were reported in *Enterobacter cloacae* in Sri Lanka [12], *E. coli* in India [13], Italy [14] and Vietnam [15], and *K. pneumoniae* in Japan [16]. NDM-4 possessed increased hydrolytic activity toward carbapenems and several cephalosporins compared to NDM-1 [11]. NDM-4 with an amino acid substitution at position 130 (Met to Leu) showed increased hydrolytic activity toward carbapenems and several cephalosporins compared to NDM-1 [11].

ST15 will be an emerging high-risk multidrug-resistant clone with carbapenem-encoding genes, including *bla*_KPCs_, *bla*_NDMs_ and *bla*_OXAs_. Outbreaks caused by ST15 OXA-48 produces were reported in France and Spain [2]. When Diancourt et al. [17] developed a MLST for *K. pneumoniae* in 2005, they already detected ST15 isolates from several countries in Europe, such as Austria, France, Portugal and Poland, and the most of the isolates were resistant to ceftazidime and ciprofloxacin. ST15 *K. pneumoniae* isolates were reported to spread in medical settings in 2005 in Hungary [2]. ST15 *K. pneumoniae* isolates were detected in other European countries, including Bulgaria, Croatia, Czech Republic, Denmark, Hungary, Italy, Netherlands, and Spain [2]; they were also detected in Asian countries, including China, South Korea, Malaysia, Singapore, Thailand, and Vietnam [2]; in African countries, including Côte d’Ivoire, Madagascar, Morocco, and Senegal [18]. These ST15 isolates frequently produced ESBL, including CTX-M, SHV-28 and TEM variants [19], and moreover, they became to produce various carbapenemases, including KPCs, OXA-48, NDMs and VIM-4 [19]. One of the well-recognized high-risk clones is CC258 which is frequently associated with KPCs-producing *K. pneumoniae* known as a high-risk clone [19], and these isolates were reported many countries, such as the United States, Greek, Norway, Sweden, Italy, Poland, Canada, Brazil and Korea [19]. ST11, a related clone ST258, was reported in KPCs-producing isolates mainly in China, but also in NDMs-producers from Czech Republic, Switzerland, Thailand, Australia, the United States, the United Arab Emirates and Greece [19].

Etest seems a more reliable method to measure colistin MICs than broth microdilution method [20, 21]. In the present study, 11 isolates were resistant to colistin with MICs 4–32 mg/L by broth microdilution method, although none of the isolates had colistin-resistant factors. Our previous study indicated that *Enterobacteriacae* isolates showed lower colistin MICs by Etest than by broth microdilution method [20]. It is necessary to find feasible susceptibility testing methods of determining the MICs of polymyxins for clinical laboratories.

## Conclusions

This study showed that carbapenem-resistant *K. pneumoniae* isolates belonged to international clones spread, and that these isolates harbored genes encoding various combinations of carbapenemases and 16S rRNA methylases, in a medical setting in Vietnam.

## Abbreviations

ESBLs: extended-spectrum β-lactamases
MBLs: Metallo-β-lactamases
MICs: minimal inhibition concentration
MLST: Multilocus sequence typing
